# Workshop on Treatment of and Postexposure Prophylaxis for *Burkholderia pseudomallei* and *B. mallei* Infection, 2010

**DOI:** 10.3201/eid1812.120638

**Published:** 2012-12

**Authors:** Rebecca Lipsitz, Susan Garges, Rosemarie Aurigemma, Prasith Baccam, David D. Blaney, Allen C. Cheng, Bart J. Currie, David Dance, Jay E. Gee, Joseph Larsen, Direk Limmathurotsakul, Meredith G. Morrow, Robert Norton, Elizabeth O’Mara, Sharon J. Peacock, Nicki Pesik, L. Paige Rogers, Herbert P. Schweizer, Ivo Steinmetz, Gladys Tan, Patrick Tan, W. Joost Wiersinga, Vanaporn Wuthiekanun, Theresa L. Smith

**Affiliations:** Author affiliations: Department of Health and Human Services, Washington, DC, USA (R. Lipsitz, J. Larsen, L.P. Rogers); National Institutes of Health, Bethesda, Maryland, USA (S. Garges, R. Aurigemma); IEM, Research Triangle Park, North Carolina, USA (P. Baccam); Centers for Disease Control and Prevention, Atlanta, Georgia, USA (D. Blaney, J.E. Gee, M. Morrow, E. O’Mara, N. Pesik, T. Smith); Royal Darwin Hospital, Casuarina, Northern Territory, Australia (A. Cheng, B.J. Currie); Menzies School of Health Research, Casuarina, (A. Cheng, B.J. Currie); Monash University Melbourne, Victoria, Australia (A. Cheng); Alfred Hospital, Melbourne (A. Cheng); Mahosot Hospital, Vientiane, Laos (D. Dance); University of Oxford, Oxford, UK (D. Dance); Mahidol University, Bangkok, Thailand (D. Limmathurotsakul, S.J. Peacock, V. Wuthiekanun); Townsville Hospital, Townsville, Queensland, Australia (R. Norton); University of Cambridge, Cambridge, UK (S. Peacock); Colorado State University, Fort Collins, Colorado, USA (H.P. Schweizer); University of Greifswald, Greifswald, Germany (I. Steinmetz); DSO National Laboratories, Singapore (G. Tan); Genome Institute of Singapore, Singapore (P. Tan); Duke–National University of Singapore Graduate Medical School, Singapore (P. Tan); and Academic Medical Center, Amsterdam, the Netherlands (J. Wiersinga)

**Keywords:** Burkholderia pseudomallei, melioidosis, Burkholderia mallei, glanders, drug therapy, postexposure prophylaxis, ceftazidime, carbapenems, trimethoprim/sulfamethoxazole, combination, amoxicillin/potassium clavulanate, clavulanic acid bacteria, antibiotic, antibacterial drugs, antimicrobial drugs, bacteria, *Suggested citation for this article*: Lipsitz R, Garges S, Aurigemma R, Baccam P, Blaney DD, Cheng AC, et al. Workshop on treatment of and postexposure prophylaxis for *Burkholderia pseudomallei* and *B. mallei* infection, 2010. Emerg Infect Dis [Internet]. 2012 Dec [*date cited*]. http://dx.doi.org/10.3201/eid1812.120638

## Abstract

The US Public Health Emergency Medical Countermeasures Enterprise convened subject matter experts at the 2010 HHS *Burkholderia* Workshop to develop consensus recommendations for postexposure prophylaxis against and treatment for *Burkholderia pseudomallei* and *B. mallei* infections, which cause melioidosis and glanders, respectively. Drugs recommended by consensus of the participants are ceftazidime or meropenem for initial intensive therapy, and trimethoprim/sulfamethoxazole or amoxicillin/clavulanic acid for eradication therapy. For postexposure prophylaxis, recommended drugs are trimethoprim/sulfamethoxazole or co-amoxiclav. To improve the timely diagnosis of melioidosis and glanders, further development and wide distribution of rapid diagnostic assays were also recommended. Standardized animal models and *B. pseudomallei* strains are needed for further development of therapeutic options. Training for laboratory technicians and physicians would facilitate better diagnosis and treatment options.

As of 2010, the literature did not contain broadly developed consensus recommendations for melioidosis therapy and postexposure prophylaxis (PEP) that could inform US government preparedness activities. The Public Health Emergency Medical Countermeasures Enterprise convened the 2010 HHS *Burkholderia* Workshop to generate expert consensus recommendations for use during a public health emergency. This enterprise is a coordinated interagency effort that is responsible for defining and prioritizing requirements for public health emergency medical countermeasures, focusing research, development, and procurement activities on the identified requirements, and establishing deployment and use strategies for medical countermeasures in the Strategic National Stockpile.

A comprehensive literature review revealed consensus recommendations for other biological threat pathogens that served as a template for recommendations made during the workshop. Use of these *Burkholderia* recommendations will improve US government efforts in preparing for public health emergencies as well as assist clinicians in case management of melioidosis. This workshop hosted internationally recognized leaders in the field of *Burkholderia* spp. research and diagnostics and eminent clinicians whose expertise in the treatment for endemic melioidosis is unparalleled. The results of the workshop were achieved through structured dialogue and question-and-answer sessions. The workshop recommendations stem largely from clinical experience with melioidosis. However, the workshop participants noted that although *Burkholderia mallei* is sensitive to gentamicin and macrolides (in contrast to *B. pseudomallei*), the recommended treatment regimens and PEP for melioidosis were considered to also be appropriate for glanders. The US government will consider these expert recommendations when developing its formal policies.

## Review of Current Knowledge

### Natural Routes of Exposure

Studies of melioidosis, the disease caused by *B. pseudomallei*, indicate that there are 3 primary routes of exposure leading to human disease in the areas of Southeast Asia and northern Australia, where the organism is endemic: inoculation, inhalation, and ingestion. The general view, based on anecdotal evidence, is that percutaneous inoculation is probably the most frequent route for natural infection, since most persons who acquire melioidosis, e.g., agricultural workers, have close and regular contact with soil (*1*). 

The proportion of melioidosis cases that result from inhalation remains speculative. Data demonstrating a rise in cases of pneumonic melioidosis in Australia during extreme weather events, which can cause the bacteria in the soil to become aerosolized, suggest that inhalation could be a major route of transmisson. The role of ingestion as a route of infection is supported by evidence of melioidosis in grazing animals after consumption of contaminated water (*2*).

### Risk Factors for Humans

The incidence of melioidosis among humans is strongly correlated with specific risk factors. The peak in incidence of natural infection occurs among adults 40–60 years of age who have underlying illness. Diabetes mellitus is the major risk factor for disease; <50% of all case-patients have diabetes as an underlying condition (3,4). Excessive alcohol consumption, chronic renal failure, and chronic lung disease are also independent risk factors. HIV infection does not appear to be a risk factor. There is also evidence for other risk factors for disease occurrence, such as malignancies and cystic fibrosis, but these disease associations are not as well established. There is no evidence that disease occurs sooner in patients with these underlying risk factors.

### Susceptibility to *Burkholderia* spp. from a Large Release

Concern over the potential for a large, deliberate release of *B. pseudomallei* and *B. mallei* arises from the fact that these pathogens were considered for or used as weapons during the 19th and 20th centuries (5). They are currently listed on the Select Agents and Toxins list compiled by the US Centers for Disease Control and Prevention, having been determined to have the potential to pose a severe threat to human and animal health.

Evidence from Australia indicates that deaths of previously healthy persons caused by melioidosis are rare (6). However, the dose of bacteria received during an intentional release could be much higher than that received during a naturally acquired infection. Although disease would probably develop rapidly in a susceptible person exposed to an aerosol release, the relationship between the dose and the probability of developing disease and its effect on disease severity are not well established and further study is needed. In a deliberate release situation, persons would be most likely to seek treatment at an acute-care facility or emergency department. This likelihood leads to the need for training and informational products created for health care professionals in these settings.

### Clinical Manifestations of Melioidosis

Clinical manifestations of naturally occurring melioidosis vary widely and can include pneumonia with or without septicemia or a localized infection involving the skin and soft tissue organs. In naturally occurring cases, pneumonia is the most common manifestation; a large inhalational exposure would probably result in pneumonia-like illness (*7*). Chronic disease might also occur; symptoms can mimic those of tuberculosis, and it is clinically challenging to distinguish these 2 diseases (*8*). When they enter care, 85% of persons with melioidosis have acute symptoms and have been symptomatic for <2 months. Chronic melioidosis infections account for 11% of clinical cases; the remaining 4% of cases are caused by reactivation of disease (*9*). At initial clinical evaluation, 55% of patients have positive blood culture results and 21% have septic shock. The death rate for sepsis-associated melioidosis is 50%–90%.

### Incubation Period

The incubation period for melioidosis can be quite variable. The exact incubation period is difficult to ascertain because it can be unclear or not known when the exposure occurred; data in this research area are extremely limited. In a study of 52 cases, the incubation period averaged 9 days (range 1–21 days) (*10*).

Although it is uncommon, some reports indicate an incubation period as long as several decades. The longest known period between exposure and clinical infection is 62 years in a World War II veteran (*11*). Based on the time to onset of symptoms of infection in persons in whom melioidosis develops after near-drowning experiences, evidence of a dose-dependent correlation between bacterial exposure and the incubation period exists, associating higher doses with shorter incubation periods (*12*).

### Diagnosis of Melioidosis

Microbiological culture remains the standard method for the diagnosis of melioidosis, but this method is not optimal because the sensitivity may be as low as 60% (*13*). Culture specimens should be obtained from blood, throat, urine, respiratory secretions, pus, and surface lesions as appropriate, for all patients with suspected cases. Any positive culture is considered diagnostic for melioidosis because *B. pseudomallei* is not considered to be a member of the colonizing microbiota. PCR to detect *B. pseudomallei* and *B. mallei* in clinical samples has been described, but is less sensitive than culture (*14*,*15*).

Gram staining and examination under light microscopy of clinical specimens or bacterial colonies can identify gram-negative bacilli with a granular or safety pin appearance, but this appearance is not specific for *B. pseudomallei*. Gram-negative organisms that are oxidase positive, gentamicin- and colistin-resistant, and susceptible to amoxicillin/clavulanic acid, also known as co-amoxiclav, should be strongly suspected to be *B. pseudomallei.* During a public health emergency, or where *B. pseudomallei* may be of high prevalence in the community, a selective medium such as Ashdown agar (*16*) that will suppress overgrowth of normal microbiota in nonsterile specimens (e.g., throat swabs) should be used for suspected cases. However, rare gentamicin-susceptible *B. pseudomallei* isolates exist (1 in 1,000), which will not grow on Ashdown agar (*17*).

Commercial *B. cepacia* agar is a good alternative if Ashdown agar is not available (*18*). Bacterial colonies are usually visible after 24 h. Colonies grown on Ashdown agar might have a violet or purple rugose, cornflower-like appearance. Colonies on nonselective media can vary in appearance and can be rugose or smooth. After positive identification is established, isolates should be collected for strain characterization and epidemiologic information as well as to aid forensic data investigations.

Biochemical tests such as the API 20NE (bioMérieux Inc., Durham, NC, USA) and Vitek II (bioMérieux Inc.) might help with confirmation, but misidentifications by both systems have been reported (*19*,*20*). Bacterial identification methods that use assays with polyclonal or monoclonal antibodies against *B. pseudomallei* and *B. mallei* have been described, e.g., a direct immunofluorescence assay that can be applied directly to clinical specimens and has been reported to have a sensitivity of 66% (*21*) and a latex agglutination assay that can be used to identify colonies (*20*). Both of these assays use in-house reagents developed in Thailand that are not commercially available but would be useful during a public health emergency for which there is a need to quickly distinguish between melioidosis and other illnesses that produce similar signs and symptoms.

Time to positive culture result (which is correlated with the level of bacteremia) is a prognostic indicator of death. Melioidosis results in a mortality rate of 74% if blood cultures show positive results within 24 h, compared with 41% mortality rate if >24 h pass before blood cultures become positive (*22*).

### Melioidosis Treatment

#### Empiric Treatment

The diagnosis of melioidosis cannot be made definitively on clinical evaluation alone because melioidosis can cause a wide variety of non-unique clinical manifestations. Clinicians are challenged to determine clinically whether a person has melioidosis or another bacterial infection requiring a different class of antimicrobial drugs for treatment. Ambiguous clinical signs and symptoms and misdiagnosis could lead to ineffective empiric treatment because of the resistance of *B. pseudomallei* to many standard antimicrobials. After the sentinel cases are identified and *B. pseudomallei* is detected in clinical specimens, many persons with systemic disease would probably be treated empirically because of limited diagnostic capabilities.

#### Antimicrobial Use and Drug Efficacy Studies

Treatment for melioidosis consists of an initial intravenous intensive phase (Table 1) that lasts for 10–14 days (or longer when clinically indicated) and an oral eradication phase (Table 2). The oral antimicrobial eradication phase substantially lowers the risk for relapse that can occur with intravenous antimicrobial drugs only. In addition to the availability of appropriate antimicrobials, access to intensive care facilities has been cited as a critical contributing factor to successful outcomes. The lack of widespread intensive care facilities in Thailand is thought to be a substantial factor contributing to a mortality rate that is on average 30% higher than that in Australia (*23*).

**Table 1:** Workshop results for initial intensive-phase therapy for *Burkholderia pseudomallei* and *B. mallei* infections during a public health emergency, 2010*

Patient	Drug	Dosage/route	Frequency
With no complications	Ceftazidime	50 mg/kg /(up to 2 g) intravenous	Every 8 h, or 6 g/d by continuous infusion after a 2-g bolus
With neuromelioidosis or persistent bacteremia or in intensive care unit	Meropenem	25 mg/kg /(up to 1 g) intravenous	Every 8 h

*Duration of intensive therapy is generally 10–14 d; however, >4 weeks of parenteral therapy may be necessary in cases of more severe disease, e.g., septic shock, deep seated or organ abscesses, extensive lung disease, osteomyelitis, septic arthritis, or neurologic melioidosis. Consider adding trimethoprim/sulfamethoxazole for patients with severe infection involving the brain, prostate, or other privileged site (same dosing as described for eradication therapy below. Can be administered by intravenous infusion over 30–60 min every 12 h, or nasogastric, or oral, as appropriate). If trimethoprim/sulfamethoxazole is included, continue for the entire duration of the intensive phase. Switching to meropenem is indicated if patient condition worsens while receiving ceftazidime, e.g., organ failure, development of a new focus of infection during treatment, or if repeat blood cultures remain positive. Depending on the severity of infection, the dose for patients >3 mo can be <40 mg/kg/; not to exceed 2 g/dose.

**Table 2:** Workshop results for oral eradication-phase therapy for *Burkholderia pseudomallei* and *B. mallei* infections during a public health emergency, 2010*

Drug	Patient characteristics	Recommended dosage/frequency
Trimethoprim/sulfamethoxazole†	Adult, >60 kg	160 mg/800 mg tablets: 2 tablets every 12 h
	Adult, 40–60 kg	80 mg/400 mg tablets: 3 tablets every 12 h
	Adult, <40 kg	160 mg/800 mg tablets: 1 tablet every 12 h OR
		80 mg/400 mg tablets: 2 tablets every 12 h
	Child	8 mg/40 mg/kg; maximum dose 320 mg/1,600 mg every 12 h
**OR**		
Amoxicillin/clavulanic acid (co-amoxiclav)	Adult, >60 kg	500 mg/125 mg tablets: 3 tablets every 8 h‡
	Adult, <60 kg	500 mg/125 mg tablets: 2 tablets every 8 h‡
	Child	20 mg/5 mg/kg every 8 h; maximum dose 1,000 mg/250 mg every 8 h

*Recommended duration of therapy is a minimum of 12 weeks. †If the organism is susceptible and the patient does not have a documented allergy to it, oral trimethoprim/sulfamethoxazole is the agent of first choice. If the organism is resistant to trimethoprim/sulfamethoxazole or the patient is intolerant, the second-line choice is co-amoxiclav. Co-amoxiclav is available in different ratios and formulations, depending on the source country. Co-amoxiclav at a ratio of 4:1 is preferred to ensure there is sufficient clavulanate (47). Preparations of co-amoxiclav are available in the United States, with ratios of amoxicillin to clavulanic acid ranging from  2:1 to 16:1, as follows: 22:1 (Augmentin 250 mg), 4:1 (Augmentin 125 mg and 250 mg suspension, Augmentin 125 mg and 250 mg chewable tablet, Augmentin 500 mg.), 7:1 (Augmentin 200 mg and 400 mg suspension, Augmentin 400 mg chewable tablet, Augmentin 875 mg oral tablet), 14:1 (Augmentin ES-600, Amoclan 600 mg suspension) and 16:1 (Augmentin XR). ‡Weight-based dosage based on 20 mg/5 mg/kg/dose.

#### Antimicrobial Drug Resistance

*B. pseudomallei* is naturally resistant to many antimicrobial drugs, and this resistance must be taken into account when selecting the appropriate treatment for the intensive phase and the eradication phase. Classes of antimicrobial drugs that are generally unsuitable for treatment include early generation β-lactams, aminoglycosides, macrolides, and fluoroquinolones. *B. mallei* has a similar resistance profile with the exception of macrolides and aminoglycosides. Primary ceftazidime resistance is a rare naturally occurring event (<1%), but this frequency may be underestimated (*24*). Carbapenems are least susceptible to the naturally occurring, chromosomally encoded β-lactamase from *B. pseudomallei* (*25*). No reports of carbapenem-resistant *B. pseudomallei* cases have been published.

#### Intensive-Phase Treatment

First-line therapy during the initial intravenous intensive phase of treatment is usually a regimen of ceftazidime or a carbapenem (either meropenem or imipenem). The practice of adding trimethoprim/sulfamethoxazole (TMP/SMX) to this phase of treatment ceased in most centers after evidence was published that addition of TMP/SMX does not decrease the mortality rate or reduce the rate of relapse, although it continues to be used by some physicians in specific circumstances, such as in patients with neurologic, prostatic, bone, or joint melioidosis (*26*). The consensus is that ceftazidime alone is adequate for the intravenous phase in the majority of cases (barring treatment failure, see below). Meropenem is also highly active against *B. pseudomallei* and should be considered as an alternative to ceftazidime. However, meropenem is associated with high costs and poor stability at ambient temperature. In addition, there are concerns that the widespread use of meropenem might lead to increased antimicrobial resistance in gram negative bacteria in general. It remains to be determined whether meropenem is superior to ceftazidime in severe melioidosis where state-of-the-art intensive care management is available.

Imipenem is an alternative carbapenem; however, only meropenem was incorporated into the consensus recommendations because of a higher incidence of side effects associated with imipenem (central nervous system adverse effects such as confusion, myoclonic activity, and seizures in up to 6% of patients) and problems of imipenem use in patients with impaired renal function.

Other antimicrobial drugs for the intravenous phase of therapy were considered but not deemed appropriate for inclusion in the consensus recommendations. Amoxicillin/clavulanic acid has been used for acute-phase treatment for melioidosis, but it requires frequent dosing and has been associated with a higher treatment failure rate than ceftazidime (*27*). Although it may have a role in empirical treatment for sepsis of unknown etiology, co-amoxiclav was not determined to have a role in the intravenous treatment for suspected or confirmed melioidosis unless no other appropriate agents are available. Other antimicrobial drugs such as ertapenem (*28*,*29*), doripenem (*29*), ticarcillin/clavulanate (*30*), and piperacillin/tazobactam (*31*) have good in vitro efficacy, but their clinical use has yet to be established. Ceftriaxone has moderate in vitro activity (*32*), but there is some evidence that it is less effective than ceftazidime and co-amoxiclav for treating melioidosis (*33*).

The duration of the intensive phase is variable, in part because symptom improvement can be slow and gradual (slower than that observed during the treatment for other infections). In Australia and Thailand, the typical duration of intensive therapy for melioidosis is 10–14 days. However, it is not unusual, especially in severe cases (e.g., septic shock, deep-seated or organ abscesses, extensive lung disease, osteomyelitis, septic arthritis, or neurologic melioidosis), for intravenous therapy to be extended to 4 weeks, or longer as necessary. The principles of treatment for sepsis and associated organ dysfunction are beyond the scope of this report but are crucial to determining early outcomes. The principles include systems to enable early identification and management of critically ill patients, careful clinical assessment and close monitoring, early stabilization of circulation and oxygenation, and timely source control with early administration of antimicrobial drugs after culture (*34*).

The workshop participants agreed that patient monitoring during treatment is crucial and made recommendations to that end. Patients with blood cultures positive for *B. pseudomallei* should repeat blood cultures every week after the start of antimicrobial therapy until negative. A repeat positive blood culture after >1 week of antimicrobial therapy is indicative of treatment failure, as is deterioration of clinical condition, e.g., worsening sepsis with organ dysfunction after 48 h of therapy. Patients whose treatment fails should be investigated for the presence of undrained abscesses, and a change from ceftazidime to meropenem might be considered. If the patient has persistent bacteremia and is already being treated with a carbapenem drug, consideration should be given to the addition of TMP/SMX at dosing described for oral eradication-phase therapy, if the patient is tolerating oral intake. Treatment failure is occasionally related to the emergence of antimicrobial drug resistance. Repeat positive cultures from samples other than blood (sputum, throat swab, urine, and pus) do not have the same clinical role for disease progression.

Discontinuation of the initial intravenous intensive phase and initiation of the oral eradication phase of treatment are indicated on the basis of clinical improvement of the patient, e.g., cessation of fever in conjunction with negative blood cultures. Guidance to clinicians during a public health emergency should emphasize that symptoms of *B. pseudomallei* infections resolve in a much slower manner than that which is usually seen with other bacterial infections treated in hospitals. The average time for fever resolution is 9 days. However, fever fluctuation may continue for as long as 1 month (*3*). Clinicians should be aware that a lack of marked improvement within 24 h of initiating antimicrobial therapy is not uncommon and in itself, does not warrant switching or changing the antimicrobial therapy. A summary of intravenous intensive-phase treatment consensus recommendations is provided in Table 1.

#### Eradication-Phase Treatment

After receiving the intensive phase of therapy, melioidosis patients require an extended period of oral antimicrobial therapy for a minimum of 12 weeks. *B. pseudomallei* is a facultative intracellular pathogen that can evade host mechanisms for clearance; without long-term therapy, patients have a high risk of relapse and development of serious disease with similar mortality rates as those for primary disease. The relapse rate after the full eradication regimen is ≈10% but rises to 30% if the oral therapy is taken for <8 weeks (*35*,*36*). Other analyses have suggested that failure to complete a minimum of 12 weeks of therapy is the major determinant of relapse (*37*).

For the eradication phase of treatment, given orally, TMP/SMX is the first-line drug, a designation based on clinical efficacy. TMP/SMX is given alone in Australia but has been given with doxycycline in Thailand. Unpublished results shared during the VIth World Melioidosis Congress from a comparative trial showed that the addition of doxycycline to TMP/SMX did not provide statistically relevant improved efficacy over TMP/SMX alone (Chetchotisakd, et al., pers. comm). The use of TMP-SMX alone is also supported by observational data (38).

TMP/SMX is associated with side effects that warrant special consideration if used in a public health emergency. These include frequent mild allergic reactions as well as some less frequent but more serious side effects such as Stevens-Johnson syndrome, bone marrow suppression, renal failure, and liver damage. TMP/SMX can cause hyperkalemia and increased serum creatinine levels, especially in patients with underlying renal impairment. Complete blood counts along with assessment of kidney function and blood electrolyte levels should be performed frequently (weekly during the first 2–3 weeks, biweekly thereafter) for patients receiving TMP/SMX. The moderately long TMP/SMX regimen recommended can cause bone marrow suppression, and it is recommended that patients who might be folate deficient be given folate supplements.

Studies have indicated that TMP/SMX might lead to adverse pregnancy outcomes (39); thus, co-amoxiclav is recommended as an alternative eradication-phase antimicrobial therapy for pregnant women. Co-amoxiclav is also recommended for those who cannot tolerate TMP/SMX. However, treatment with co-amoxiclav or doxycycline has been associated with a higher rate of relapse compared with TMP/SMX therapy (*40*). Pharmacokinetic studies suggest that more frequent administration than that of standard regimens might be needed to ensure adequate levels of clavulanic acid (*41*). The consensus therapy recommendations for eradication-phase treatment of patients with melioidosis during a public health emergency are detailed in Table 2.

#### Postexposure Prophylaxis 

Consensus guidance on PEP in the context of laboratory exposure has been published (*42*). Owing to the paucity of data on PEP efficacy, these recommendations have changed little since the 2008 publication; current recommendations are shown in Table 3. The recommended antimicrobial drugs include TMP/SMX and co-amoxiclav.

**Table 3:** Workshop results for postexposure prophylaxis for *Burkholderia pseudomallei* and *B. mallei* infections during a public health emergency, 2010*

Drug	Patient characteristics	Recommended dosage/frequency
Trimethoprim/sulfamethoxazole	Adult, >60 kg	160 mg/800 mg tablets: 2 tablets every 12 h
	Adult, 40–60 kg	80 mg/400 mg tablets: 3 tablets every 12 h
	Adult, <40 kg	160 mg/800 mg tablets: 1 tablet every 12 h OR
		80 mg/400 mg tablets: 2 tablets every 12 h
	Child	8 mg/40 mg/kg; maximum dose 320 mg/1,600 mg every 12 h
**OR**		
Amoxicillin/clavulanic acid (co-amoxiclav)		
	Adult, >60 kg	500 mg/125 mg tablets: 3 tablets every 8 h†
	Adult, <60 kg	500 mg/125 mg tablets: 2 tablets every 8 h†
	Child	20 mg/5 mg/kg every 8 h; maximum dose 1,000 mg/250 mg every 8 h

*Duration of post-exposure prophylaxis is 21 d. If the organism is susceptible and the patient does not have a documented allergy to it, oral trimethoprim/sulfamethoxazole is the agent of first choice. If the organism is resistant to trimethoprim/sulfamethoxazole or the patient is intolerant, the second-line choice is co-amoxiclav. †Weight-based dosage based on 20 mg/5 mg/kg/dose.

Recommended duration of PEP is 21 days, based on the premise that this regimen would provide prophylaxis covering the common range of incubation periods (43). Animal data that further characterize the incubation period might provide additional data on which to base the PEP duration, but current data are scarce.

Providing PEP to all persons potentially exposed in a large exposure event is problematic because it is not possible to distinguish those who were exposed but are asymptomatic from those who were not exposed. Currently available serologic diagnostic tools would not be useful for assessing exposure immediately after the event. The potential benefit of PEP must be weighed against the potential of the first-line recommended drug, TMP/SMX, to cause severe adverse effects. Devising a policy regarding PEP for persons in a large exposed area could be difficult, considering current weaknesses in both diagnosis and treatment options.

#### Animal Studies/PEP

A paucity of animal data regarding PEP complicates informed PEP recommendations. The studies that have been conducted lack consistency in nearly all aspects of study design, including variability in the strain of *B. pseudomallei* used, the preparation of the inoculum, the inoculating bacterial dose, the route of bacterial challenge, the route of antimicrobial drug treatment, the frequency of drug administration, the study conduct (conducting a treatment study when a PEP study was the goal), and the timing of the commencement of antimicrobial therapy. Only 1 research group has demonstrated sufficient consideration of whether the dose of antimicrobial drug given was adequate (i.e., the duration over which the drug was maintained above the MIC for the antibacterial drug combination) (*44**,**45*). In addition, animal studies thus far have not used a consistent route of exposure. Ideally, the route of exposure used in animal studies regarding PEP should reflect the route of exposure that would most likely occur during a public health emergency, modeling the scenario where PEP would be needed for large numbers of patients.

## Conclusions

There is a need for the scientific community to agree to a common set of strains for melioidosis studies because there are large variations in strain virulence and ensuing disease pathogenesis. Strains K96243 or 1026b have been used. Once consensus is reached, strains should be stored and distributed by a repository such as the Biodefense and Emerging Infections Research Resources Repository (www.beiresources.org). Furthermore, consensus is needed to determine which animals are most suitable for use in melioidosis studies. Most animal studies to date have involved mice (inbred and outbred), hamsters, and rats, and it is unclear which combination of animals and organism strain best represents human disease. The use of larger mammals, including nonhuman primates, should be considered when designing PEP efficacy studies. Currently, there is a lack of PEP data for nonhuman primates (*46*).

The dialogue during this workshop provided much useful information that can benefit preparedness and response efforts for melioidosis and glanders. In addition, it raised compelling questions and issues regarding future research on *B. pseudomallei.*
